# On-Chip Plasmonic Slit-Cavity Platform for Room-Temperature Strong Coupling with Deterministically Positioned Colloidal Quantum Dots

**DOI:** 10.1021/acs.nanolett.5c05910

**Published:** 2026-02-27

**Authors:** Jin Qin, Benedikt Schurr, Patrick Pertsch, Daniel Friedrich, Max Knopf, Saeid Asgarnezhad-Zorgabad, Lars Meschede, Daniel D. A. Clarke, Monika Emmerling, Artur Podhorodecki, Ortwin Hess, Bert Hecht

**Affiliations:** † Nano-Optics and Biophotonics Group, Experimentelle Physik 5, Physikalisches Institut, Universität Würzburg and Röntgen Research Center for Complex Material Research, Physics Institute, Am Hubland, Würzburg D-97074, Germany; ‡ School of Physics and CRANN Institute, Trinity College Dublin, Dublin 2, Ireland; § Department of Experimental Physics, 49567 Wroclaw University of Science and Technology, Wybrzeze Wyspianskiego, Wroclaw 50-370, Poland

**Keywords:** exciton−plasmon coupling, plasmonic cavity, dielectrophoresis, quantum emitter, Stark effect

## Abstract

Strong coupling between quantum emitters and optical cavities underpins many quantum photonic technologies, yet achieving this regime at room temperature in compact, deterministic on-chip platforms remains challenging due to the difficulty of fabricating cavities with ultrasmall mode volumes and precisely positioning quantum emitters. Here, we demonstrate a robust quantum plasmonic device in which colloidal quantum dots are strongly coupled to plasmonic slit cavities. Our dielectrophoresis-based positioning technique with real-time photoluminescence feedback enables parallel device fabrication and straightforward integration with additional optical elements, such as waveguides. Our measurements reveal clear photoluminescence-resolved Rabi splitting at room temperature in precharacterized cavities, with device-to-device variations scaling with the average number of coupled quantum dots. While electrical tuning via the quantum-confined Stark effect is enabled by integrated electrodes, its impact is largely overshadowed by room-temperature spectral diffusion. These results establish a scalable and electrically addressable plasmonic platform for room-temperature quantum technologies.

## Graphical abstract

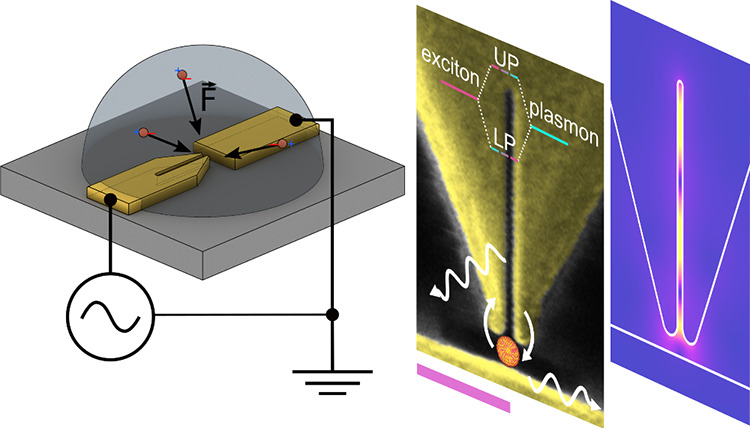

The spontaneous emission of a quantum emitter can be significantly modified by the local density of optical states when coupled to a resonant optical mode, leading to rich light–matter interaction phenomena, such as Purcell-enhanced photon emission.^1–6^ When the emitter and cavity are in the strong coupling regime, a new hybrid light–matter state, known as a polariton, emerges.^7^ This state exhibits both photonic and excitonic characteristics and has enabled advances in areas such as Bose–Einstein condensation,^8^ enhanced chemical reactivity,^9^ and potentially modified superconductivity.^10^

Spectroscopically, strong coupling manifests as Rabi splitting in scattering, absorption, or photoluminescence (PL), indicating at least one complete cycle of coherent energy exchange between the emitter and the cavity. Achieving this regime requires a coupling strength *g* that exceeds both the cavity loss and emitter decay rates, including dephasing, which is especially challenging at room temperature. Dielectric cavities, with their high quality factors (*Q*), are advantageous in terms of small loss.^11,12^ However, to maximize the spectral overlap between such high-*Q* cavity resonances and quantum emitters, a cryogenic environment is typically required, as the emitter line width broadens with an increase in temperature. The line widths of the two coupled entities should be comparable to ensure sufficient spectral overlap, thereby maintaining the strong coupling condition.

In contrast, plasmonic cavities, consisting of nanoscale metallic structures, offer a deep subwavelength confinement of optical modes with moderate *Q* factors, yielding ultrafast energy transfer. This makes them promising candidates for achieving strong coupling at room temperature. However, realizing strong coupling in such systems demands precise spatial and spectral matching between the quantum emitter and the plasmonic cavity. In particular, optimal spatial matching poses a major challenge due to the need for nanometer-scale positioning of the emitter within the localized plasmonic field. Recent advancements have demonstrated various platforms and techniques to achieve strong coupling between quantum emitters and plasmonic cavities at room temperature, including particle-on-mirror structures,^13–16^ plasmonic tips,^17,18^ scanning slit cavities,^19,20^ bowtie antennas,^21–23^ and nanoparticles.^24–27^ Methods such as scanning probes^17,18^ or our own approach of integrating a slit cavity into an AFM tip^19,20^ offer additional flexibility by leveraging the nanometer-scale precision of AFM scanners, yet such devices are inherently serial and not easily scalable. Particle-on-mirror structures, on the other hand, provide extremely small mode volumes through self-assembled junctions.^13,15^ However, most implementations rely on drop-casting techniques,^13–16,24–26^ where quantum emitters are randomly deposited onto the plasmonic structures, resulting in nondeterministic placement and challenges in characterization. Moreover, while multistack architectures have been proposed for building devices,^15,18^ a planarized design is more favorable for integration with nanocircuitry or ultracompact nanophotonic platforms, particularly when additional elements can be incorporated into the strongly coupled system to enable extended functionality, such as integration with waveguiding and grating structures. Despite the progress of these approaches, fully on-chip integration remains highly desirable because it combines robustness, scalability, and compatibility with established top-down nanofabrication methods, offering a clear pathway toward ultracompact quantum photonic devices.

Here, we develop an on-chip plasmonic platform consisting of a slit-cavity structure and a counter electrode, as illustrated in Figure 1a. This open architecture allows retrofitting with a variety of quantum emitters and, in principle, can be integrated with additional waveguides. In this sense, it serves as a prototypical structure for devices whose functionality is based on cavity quantum electrodynamic effects, particularly strong coupling. Using a dielectrophoresis (DEP) process, one or several quantum emitters can be positioned near the tip of the slit, where the localized plasmonic-cavity mode exhibits a pronounced field maximum. Split PL spectra can be observed, indicating strong coupling. We find that the Rabi splitting strength varies between devices, which we attribute to differences in the number of quantum emitters coupled to the cavity mode. Additionally, we attempt to tune the emitter resonance via the quantum-confined Stark effect (QCSE) by applying an external voltage. However, due to significant spectral diffusion of the emitters at room temperature, the tunability can barely be observed with a typical decrease in PL intensity. Our experiments demonstrate on-demand strong coupling at room temperature in an ultracompact on-chip plasmonic device, highlighting its strong potential for integration into quantum photonic circuits.

**Figure 1:**
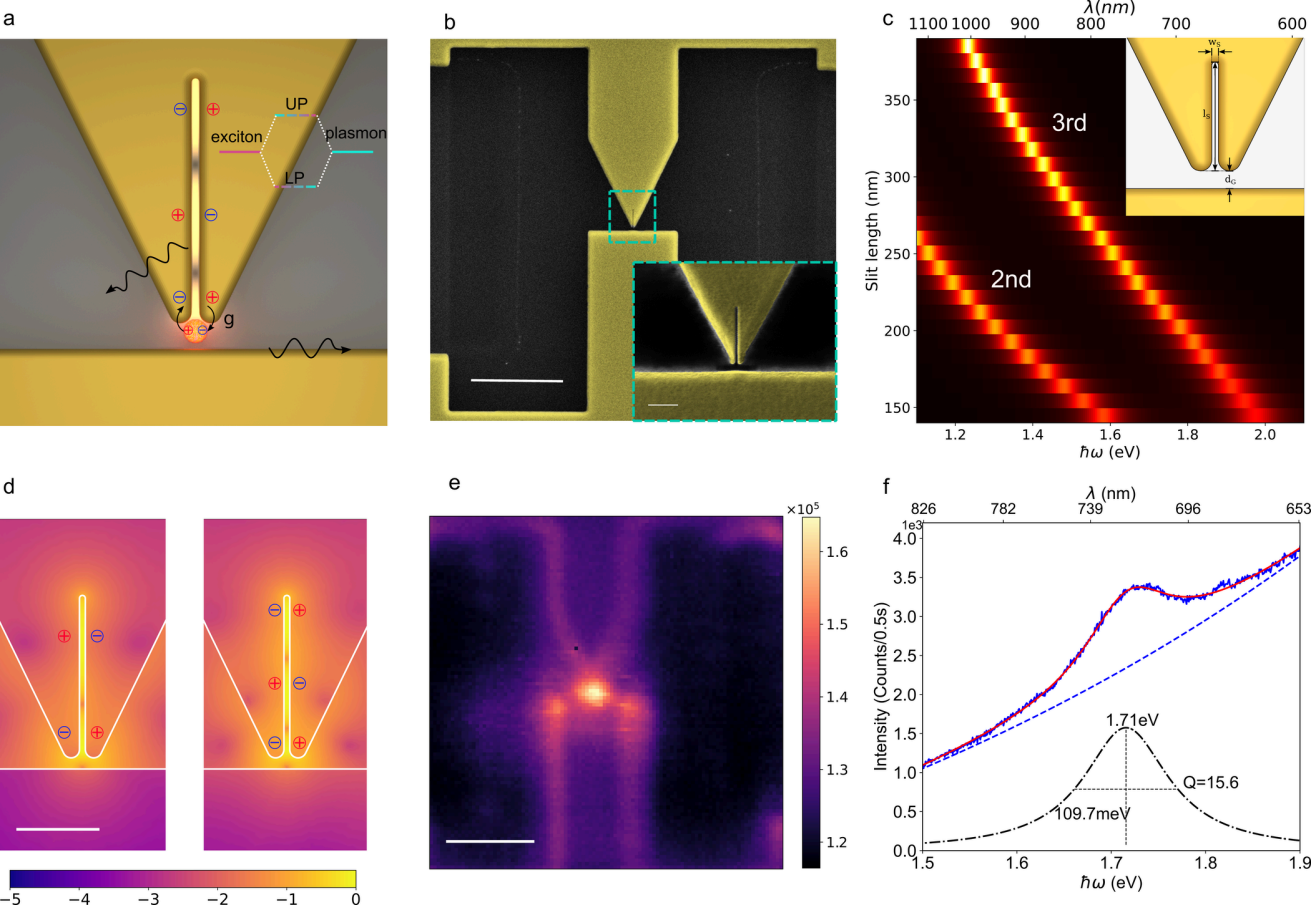
Schematic of the on-chip plasmonic strong coupling platform and characterization of the plasmonic nanoslit cavity. (a) Illustration of a single quantum emitter interacting with an on-chip plasmonic nanoslit cavity. The third-order nanoslit-cavity mode is strongly coupled to the quantum emitter. In the experiment, the emitter is first excited nonresonantly using a green laser. Due to coherent energy exchange, polaritons are formed that decay via the emission of a photon. UP denotes the upper polariton, and LP lower polariton. (b) SEM images of the on-chip strong coupling device. Scale bar: 1 μm. The inset shows a close-up view of the tip area indicated by a dashed rectangle. Scale bar: 100 nm. (c) Second- and third-order FP modes obtained through quasi-normal mode analysis (section S1) for varying slit lengths and a fixed slit width of 7 nm. (d) Electric field distributions (log scale, |*E*|^2^) of the second- and third-order FP modes in the nanoslit cavity. Scale bar: 100 nm. (e) Hyperspectral PL map recorded by scanning the excitation spot across a sample region centered on the nanoslit cavity. Notably, this PL signal can be detected only under a high laser power (wavelength of 532 nm, power of 500 μW, intensity of 4.4 × 10^9^ W/m^2^). The PL map is generated by integrating spectral intensities between 1.68 and 2.00 eV corresponding to the spectrum in panel f. Scale bar: 1 μm. (f) PL spectrum (blue line) of the cavity mode when the excitation laser is focused on the slit structure. The spectrum is fitted with a cumulative model (red line) consisting of a Lorentzian resonance (black dotted–dashed line, corresponding to the slit-cavity mode) and an exponential background (blue dashed line). From the Lorentzian fit, the cavity resonance energy and quality factor are extracted.

## Plasmonic Slit-Cavity Characterization

On-chip plasmonic slit-cavity structures are fabricated using monocrystalline gold flakes with a thickness of 40 nm, employing helium ion milling (see the scanning electron microscopy (SEM) image and inset of Figure 1b). The primary motivation for using solution-synthesized Au flakes in this work is their single-crystalline nature and ultrasmooth surface, which are difficult to achieve using standard evaporated Au films. Single-crystal Au flakes significantly reduce grain-boundary scattering and surface roughness, both of which are critical for achieving reproducible plasmonic resonances and strong field confinement in sub-10 nm slit cavities. These properties are particularly important for observing strong coupling at room temperature and for ruling out spurious spectral features arising from structural inhomogeneities. The nanoscale resolution and robustness of this fabrication method enable the on-demand creation of individual well-defined structures with high reproducibility. The slit cavity supports Fabry–Pérot (FP) modes, which depend on the slit’s length and width. As demonstrated in Figure 1c, using quasi-normal mode analysis (section S1 of the Supporting Information), the resonance of second- and third-order FP modes, characterized by the number of field maxima in the slit (Figure 1d), can be tuned by varying the slit length while keeping the width fixed. Besides, we also find that the free spectral range of the FP modes is sufficiently large to allow only a single FP mode to couple with the quantum emitter. Furthermore, the second- and third-order FP modes exhibit quadrupolar-like field distributions (Figure 1d), which helps to suppress radiation losses and therefore yield a relatively high *Q*.

In our experiment, we chose a slit length of 215 nm and a width of 7 nm to match the resonance of the quantum emitter. The slit width is comparable to the size of the emitter, enabling an effective spatial overlap of the mode emanating at the tip and the quantum emitter. An additional counter electrode is fabricated opposing the tip of the slit cavity with a gap of approximately 20 nm. This adds great versatility to the platform. For example, AC and DC voltages can be applied across the gap, allowing for dielectrophoresis and to induce Stark shifts in emitters. The resulting FP mode in the plasmonic slit cavity is verified through photoluminescence (PL) spectroscopy under high excitation intensity, allowing both spatial and spectral characterization.

To record spatially resolved PL spectra of the device shown in Figure 1b, we use hyperspectral imaging with the experimental setup depicted in Figure S2. Figure 1e shows a hyperspectral map collected by scanning the excitation spot across the entire device area. A bright localized spot appears at the plasmonic slit-cavity position when the signal is integrated over the spectral range of its resonance. The corresponding PL spectrum recorded at the brightest spot is shown in Figure 1f, where a shoulder on the broad gold PL background indicates the slit-cavity mode resonance. By applying a cumulative fit, we extract a cavity resonance at 1.71 eV with a quality factor *Q* of 15.6 (section S2).

## Quantum Emitter Characterization

The quantum emitters used in our experiments are commercial colloidal core–shell quantum dots (Qdots, 705 nm, CdSeTe/ZnS, Thermo Fisher). These Qdots are first spin-coated onto a clean coverslip to characterize their physical and optical properties. The atomic force microscopy (AFM) image in Figure 2a shows a typical single Qdot exhibiting an apparent diameter of approximately 15 nm and a height of approximately 7 nm, which closely matches the width of the slit cavity. The Qdot’s emission spectrum, excited by a green laser, is displayed in Figure 2b. It can be fitted with a Lorentzian profile, yielding a resonance energy of 1.76 eV and a relatively broad emission line width of 140 meV. Single-photon emission by individual Qdots is confirmed by intensity autocorrelation measurements, as depicted in Figure 2c. Clear antibunching behavior is observed with a dip down to 0.25 at zero time delay. The corresponding uncertainty analysis is presented in Figure S4b. Under ambient conditions, some Qdots are chemically unstable and prone to oxidation when exposed to laser excitation resulting, e.g., in a resonance shift.^20^ To verify the absence of such behavior, we record time-traced PL spectra and extract the resonance energies and line widths using Lorentzian fitting, as shown in Figure 2d and Figure S3. The data show no significant spectral shifts (i.e., no consistent blue- or red-shift), but we do observe substantial spectral diffusion at room temperature with spectral variations as large as 50 meV.

**Figure 2:**
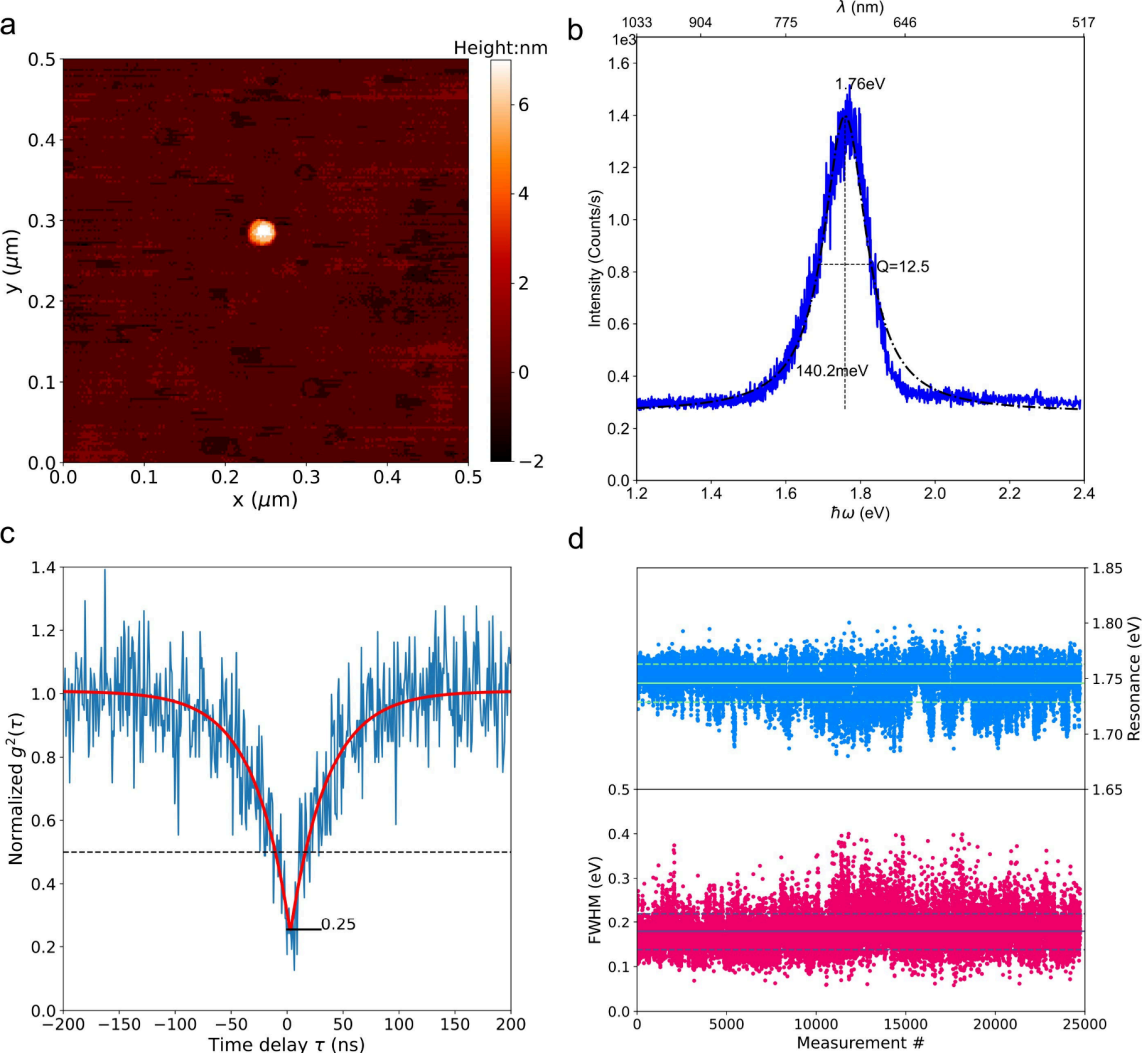
Characterization of single Qdots. (a) AFM image of a representative single Qdot, spin-coated on a glass coverslip. (b) PL emission spectrum (blue line) under green laser excitation with a power of 1 μW (intensity of 8.8 × 10^6^ W/m^2^). The spectrum is fitted with a Lorentzian function (black dotted–dashed line) to extract the resonance energy and line width. (c) Photon statistics of the Qdot emission measured by second-order autocorrelation. A clear antibunching dip down to 0.25 at zero-time delay confirms single-photon emission behavior. The corresponding uncertainty analysis is presented in Figure S4b. (d) Time-dependent PL spectra of a single Qdot (Figure S3), recorded with a temporal resolution of 33 ms. Resonance energies (blue dots) and line widths (red dots) are extracted from Lorentzian fitting. The average value and one standard deviation are indicated by solid and dashed lines, respectively.

## DEP Process

To achieve strong coupling, one of the main challenges is positioning the quantum emitter with nanometer-scale precision to ensure spatial alignment with the localized-cavity mode. To overcome this problem, we employ a DEP process with a real-time feedback mechanism to attract and position single or multiple Qdots at the tip of the slit cavity, where the cavity mode’s plasmon-mediated electric field is also concentrated.^28^ Prior to the DEP process, the Qdot solution is diluted in pure water at an appropriate ratio (1:10000). A 5 μL droplet of this diluted solution is then deposited onto the surface of the on-chip device. An alternating voltage is applied to the electrode containing the slit cavity, while the counter electrode is grounded (Figure 3a). This generates a nonuniform electric field that polarizes the quantum dots in solution, producing a net force that can either attract the Qdots toward or repel them from the slit tip, depending on the applied frequency (section S4). By carefully tuning the applied voltage and frequency, we can make the net force attractive, drawing nearby Qdots toward the slit tip, where the electric field gradient is strongest.

**Figure 3:**
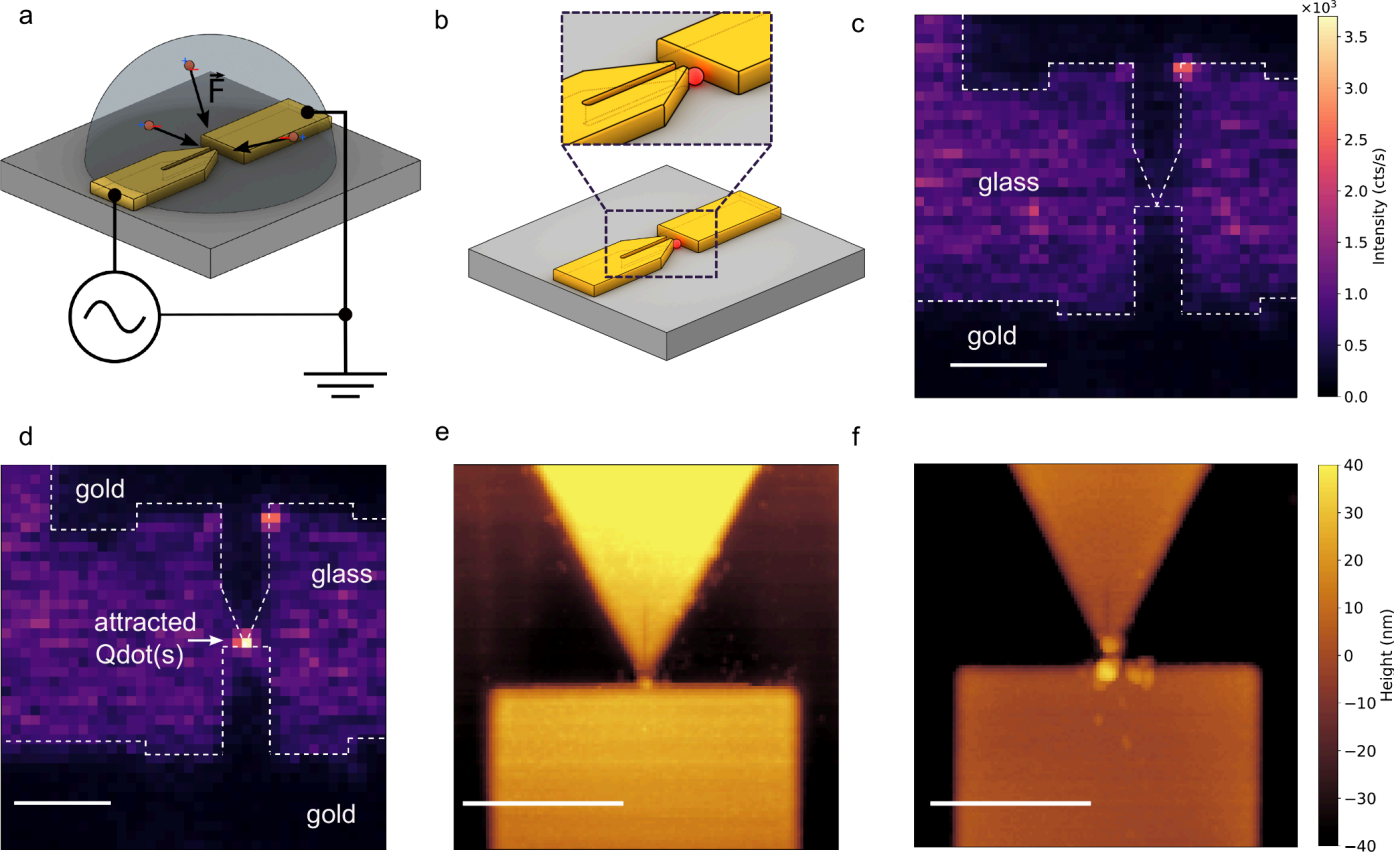
DEP process. (a) Schematic illustration of the setup used for DEP. A droplet of the Qdot solution (diluted 1:10000 with pure water) is placed on top of the device, and an AC voltage (amplitude, 2 V; frequency, 2 MHz) is applied across the plasmonic nanoslit cavity (left) and the grounded counter electrode (right). Under the influence of the nonuniform electric field, for the right range of frequencies, Qdots experience a dielectrophoretic force and migrate toward the tip of the slit cavity, where the field gradient is maximal. (b) Illustration of the resulting coupled structure after DEP. A Qdot (represented by the red sphere) is positioned at the end of the slit cavity. The inset shows a magnified image. (c and d) Real-time feedback mechanism based on PL signals detected by an APD, while scanning the focused excitation laser (power: 1 μW; intensity: 8.8 × 10^6^ W/m^2^) over the device. (c) Prior to DEP, no confined PL spot is observed. Dark regions correspond to the gold surface, and the brighter areas correspond to the weak autofluorescence of the cover glass. Also, at these positions, more excitation light is transmitted and possibly excites Qdots suspended in the solution, adding to background signals. (d) Once a Qdot is captured at the slit tip, a bright and localized PL spot appears. Scale bar: 1 μm. Panels c and d share the same color bar clearly showing an increased fluorescence at the tip apex between the electrodes. (e and f) AFM scans of two representative devices after the DEP process. (e) Single Qdot positioned at the cavity tip using DEP parameters: voltage of 2 V, frequency of 2 MHz, and duration of 3 s. (f) With modified DEP parameters (voltage of 2 V, frequency of 0.5 MHz, and duration of 3 s), a larger number of particles, possibly including contaminants, accumulate around the cavity tip. Scale bar: 500 nm.

To ensure successful placement of Qdots within the region of interest, we implement real-time feedback based on PL detection by using a high-bandwidth avalanche photodiode (APD). Before initiating the DEP process, we scan the excitation laser (532 nm, 1 μW) over the sample and record the resulting PL map (Figure 3c). From this map, the approximate position of the slit cavity can be identified, as indicated by the white dashed line. During DEP, the region of interest (slit cavity) is continuously monitored at a repetition rate of 100 Hz. As soon as Qdots are trapped at the cavity tip, a sharp increase in PL intensity is observed at that location (Figure 3d), signaling the successful placement of Qdots. At this point, the DEP process is interrupted, and the device is allowed to dry for several minutes. The Qdots remained anchored at the desired location, as shown in Figure 3b. More details about the DEP process, its optimization, and parameters used can be found in section S4.

The resulting coupled structures are first characterized using AFM. By varying the DEP parameters, such as applied frequency and voltages, we can achieve different numbers of captured Qdots. In Figure 3e, it appears that only a single Qdot is attracted; however, this observation is not conclusive, as multiple Qdots could be stacked vertically, which cannot be resolved by AFM. Additionally, the DEP process attracts not only Qdots but also sometimes contaminants, as observed in Figure 3f.

## Strongly Coupled Spectra in PL

Split PL spectra indicating strong coupling can be measured directly after the coupled structures are formed, as described above. Many experiments rely on observing split scattering spectra as evidence of strong coupling; however, such features can also arise from alternative mechanisms such as Fano resonances^22,24^ or inhomogeneous dielectric environments,^29^ making the interpretation ambiguous. In contrast, split PL spectra, when combined with careful analysis of the uncoupled states, provide more definitive evidence of strong coupling. Typically, we focus a green excitation laser (power of 1 μW) on a position that simultaneously covers both the plasmonic slit cavity and Qdots, ensuring that the Qdots are primarily excited. To identify the correct position, we recorded a hyperspectral image and located the brightest spot by integrating over the spectral range of the Qdot emission. Notably, PL signals from the bare plasmonic slit cavity can be detected only under high excitation power (500 μW), and thus, they are negligible under our low-power conditions for strongly coupling experiments.

Once the Qdots are strongly coupled to the slit cavity, polaritons are formed through coherent energy exchange between excitons and cavity plasmons. This interaction manifests spectrally as a characteristic double-peak feature in the PL spectra, as shown in Figure 4a–c, recorded from three different devices prepared in separate experiments under the same conditions.

**Figure 4:**
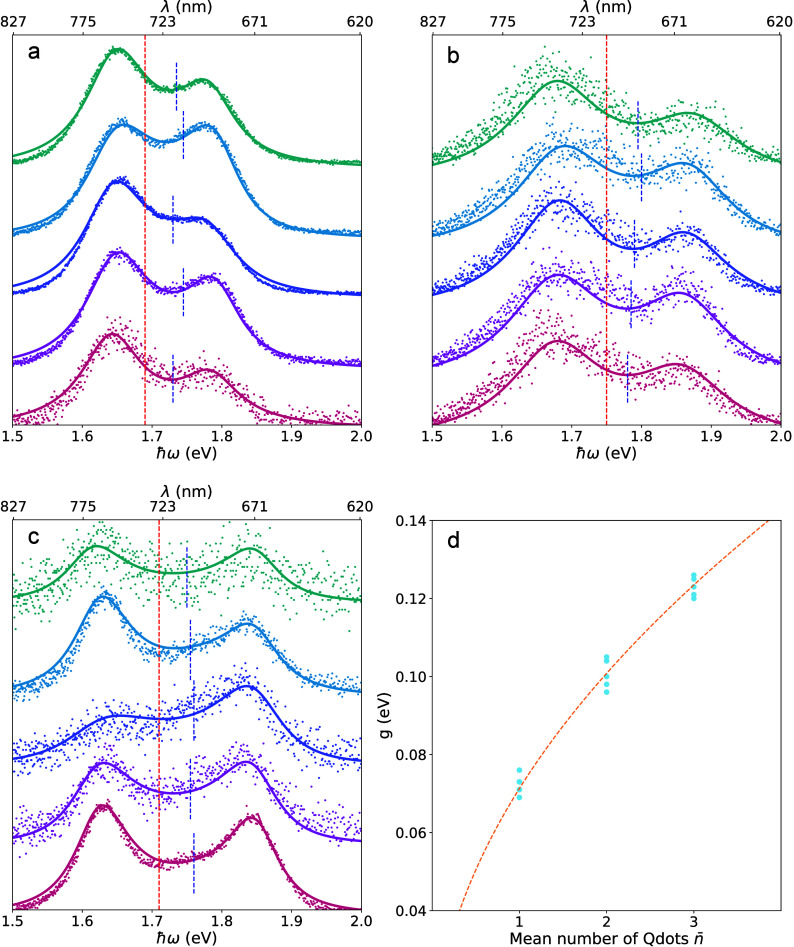
Split PL spectra recorded from different coupled structures. (a–c) Split PL spectra (dots) recorded from three different coupled structures and the corresponding fit (solid lines) of the quantum model. For each structure, five spectra were recorded with different integration times to verify its reproducibility and to elucidate the influences of spectral diffusion. Before the coupled structures were fabricated, the resonance of the slit cavity is characterized by PL with high power. The resonance frequency is marked by a red dashed line. The Qdot’s resonance is labeled by a blue dashed line, which is not fixed in the fitting to accommodate small but finite spectral diffusion at room temperature. The fitting parameters can be found in Tables S1–S3, respectively. (d) Extracted coupling strengths from three different structures are indicated by the dots. The orange line represents the corresponding comparison to theory assuming multiple emitters (indicated by *n̅*) coupled to a single cavity.

To interpret the experimental results, we use the Jaynes–Cummings Hamiltonian to model the interaction between a single plasmonic nanocavity mode and a two-level quantum emitter.^30^ Lindblad terms are incorporated into the master equation to account for incoherent pumping and all relevant loss and dephasing channels (section S6). Because each experiment begins with a characterization of the slit cavity, cavity resonance frequency ω_cav_ and loss rate γ_cav_ can be accurately determined and fixed in the model. In contrast, Qdot emission frequency ω_qd_ and line width γ_qd_ are significantly affected by spectral diffusion at room temperature (Figure 2d). These parameters are therefore adjusted slightly to fit the measured spectra (section S7).

Figure 4a presents five PL spectra acquired with different integration times along with theoretical fits using the quantum model. Variation of the integration time can provide insights into the effects of spectral diffusion. From the fits, an average coupling strength (*g*) of 72.4 ± 2.3 meV is extracted, which clearly satisfies the strong-coupling criterion: 2*g* > (γ_cav_ + γ_qd_)/2. Meanwhile, when the integration time varies from 33 ms to 1 s, the splitting feature remains clearly visible, with the Rabi splitting strength nearly unchanged.

The same fitting approach is applied to data sets recorded for different structures (Figure 4b,c), fabricated using identical procedures. The extracted coupling strengths are 101.4 ± 1.8 and 123.0 ± 2.3 meV, respectively. These variations in different coupled structures are likely to arise from different numbers of Qdots coupling to the same slit-cavity mode, with the effective coupling strength expected to scale as √ in Figure 4d, in agreement with the Tavis–Cummings model.^31^ This interpretation is supported by the fact that, in the Qdot aqueous solution, some emitters are present in the form of clusters, which can be verified by AFM scans of spin-coated Qdots on the coverslip in Figure S5 (Supporting Information S3). Additional results from other devices are presented in Figure S8d, where fluctuations in coupling strength can be ascribed to variations in the dipole orientation of individual Qdots. Furthermore, finite-difference time-domain (FDTD) simulations (Figure S7) provide an estimate of the single-emitter coupling strength of 72.8 meV, based on the calculated effective mode volume and the effective dipole moment of a Qdot. In our experiments, we did not observe coupling strengths exceeding those reported here. The reason for this is likely the extent of the spatially confined mode at the nanoslit-cavity tip, where the available mode volume restricts the number of Qdots that can couple strongly. Generally, the Qdot’s number can be measured via resolving the antibunching dip in photon statistic measurement. However, performing photon statistics measurements directly on cavity-coupled Qdots is considerably more challenging. After coupling, the polaritonic PL signal is intrinsically weak and accompanied by significant background contributions, which severely limits the signal-to-noise ratio. In addition, in the strongly coupled plasmonic system, the polariton lifetime is dramatically reduced and is expected to lie in the tens of femtoseconds regime due to the ultrafast decay of plasmonic modes (section S8). Resolving such rapid dynamics in conventional photon-correlation experiments is practically impossible, as the temporal resolution of standard single-photon counting setups is limited to hundreds of picoseconds. This limitation arises from the intrinsic timing jitter of photodetectors and their associated electronics. Consequently, any antibunching dip in the photon statistics would be narrower than the shortest possible time bin and therefore unresolvable. Recently, advances in ultrafast two-dimensional electronic spectroscopy have demonstrated significant potential for resolving the dynamics of coherent polaritonic states.^32,33^

A key advantage of our on-chip platform is the ability to deterministically characterize each slit cavity prior to coupling experiments, something not feasible with systems such as bowtie antennas or particle-on-mirror structures. This pre-characterization eliminates uncertainties due to multiple-plasmonic mode interference and enables accurate estimation of coupling strength without uncertainties due to possibly large detunings or uncoupled entities. In the future, our on-chip platform can be further integrated with optical waveguides to form functional nanocircuitry. However, waveguides fabricated from pure gold typically exhibit high propagation losses, which limit their suitability for long-distance optical routing. To address this limitation, we have recently developed Au–Ag alloy structures that combine the lower optical damping of silver with the improved chemical stability of gold, offering a potential route to extend the effective propagation length of plasmonic waveguides.^34^ In this scenario, CMOS-compatible metal deposition techniques with improved surface quality and reduced optical damping can be employed to enable scalable manufacturing. In addition, several established hybrid approaches enable efficient coupling between plasmonic nanoantennas or cavities and small-loss dielectric waveguides (e.g., Si, SiN, or TiN) through near-field or evanescent coupling over submicrometer distances. In such architectures, the plasmonic slit cavity serves as a highly localized light–matter interaction region, while optical routing is handled by adjacent dielectric components.

## Electrically Connected Strongly Coupled Device

Benefiting from the electrically connected structure and the gap between the counter electrode and the slit tip, we leverage the quantum-confined Stark effect (QCSE) to deliberately tune the resonance of the trapped Qdot by applying a strong external electric field, as illustrated in Figure 5a. QCSE is a well-known phenomenon whereby increasing the electric field reduces the overlap between the electron and hole wave functions, resulting in a decreased PL intensity and a spectral shift. Typically, the linear and quadratic dependence on the electric field is sufficient to describe frequency shifts as^35^

$$\Delta \omega =-\mu E-\frac{1}{2}\alpha E^{2}$$

where μ and α are the permanent dipole moment and polarizability of the Qdot, respectively, and *E* is the electric field acting on the Qdot, consisting of two contributions: *E*_int_, the internal electric field arising from the Qdot’s charged state (randomly oriented, e.g., through the addition or removal of a surface electron), and *E*_applied_, the externally applied electric field.

**Figure 5:**
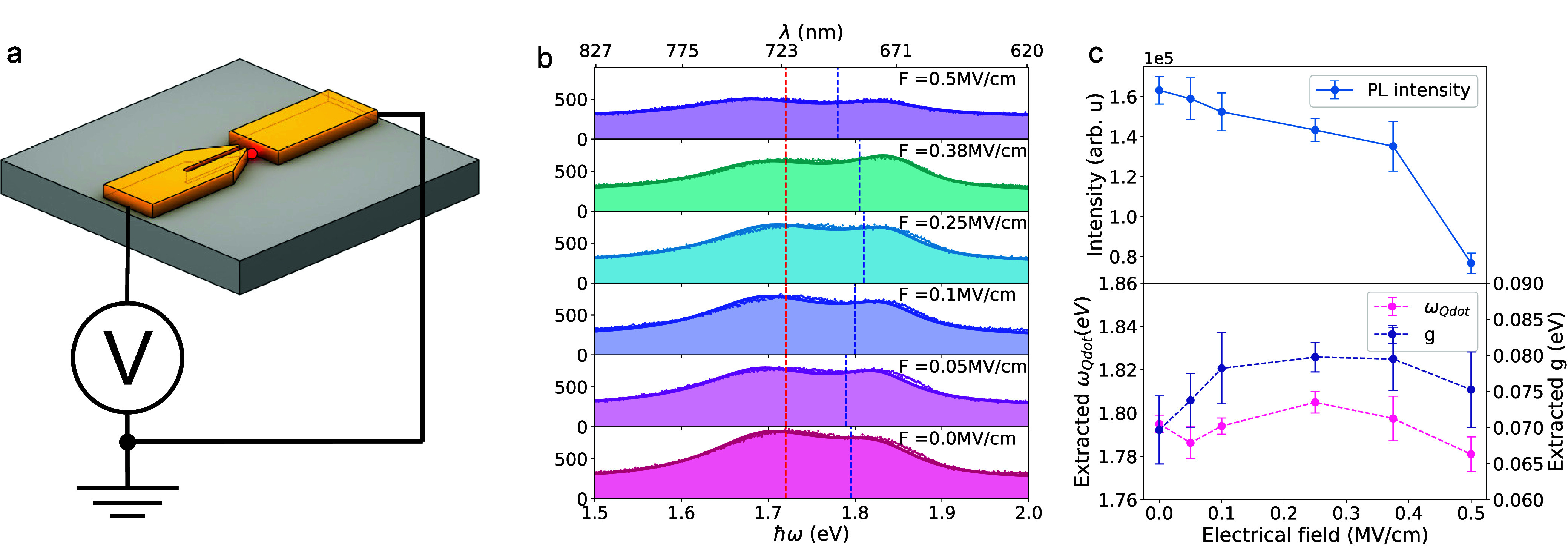
Electrically connected on-chip strong coupling devices. (a) Schematic illustration of the electrically connected on-chip strong coupling device. A DC voltage source is connected to the two electrodes after the coupled structure is formed. (b) Measured split PL spectra (dots) and corresponding fits from the quantum model (solid lines) under varying applied electric fields from 0 to 0.5 MV/cm. The red dashed line indicates the cavity resonance characterized prior to the DEP process, while the blue dashed line marks the Qdot resonance extracted from the fit. (c) Statistical analysis of PL intensities, extracted Qdot resonances, and coupling strength *g* across multiple spectra under different applied voltages. The PL intensity is calculated by integrating the total counts across the full spectral range.

In Figure 5b, we present PL spectra characteristic of strong coupling of a single Qdot to a plasmonic nanoslit cavity (Figure 5a) recorded under different applied DC voltages. When no voltage is applied, the extracted coupling strength is the same as that shown in Figure 4a for an average emitter number *n̅* = 1. As the applied field strength increases, a clear decrease in PL intensity is observed. However, a significant and consistent shift in the Qdot resonance energy is not observed. To further investigate this, we analyze multiple split PL spectra under varying applied voltages and extract both the overall PL intensity and the Qdot resonance energy from the spectra, as shown in Figure 5c. While the PL intensity consistently decreases with an increase in *E*_applied_, the resonance shift exhibits no clear dependence on the electric field, with an overall tuning range of approximately 20 meV, still within the typical range of spectral diffusion. Notably, similar fluctuations in the Qdot resonance are also observed in the absence of an applied voltage, as shown in Figure 4a–c. Similar observations are presented in section S9 and Figure S9a,b, where alternating voltages were applied to the hybrid structures. Once again, the resonance shifts show no clear dependence on the applied voltage.

The Qdot resonance shift induced by the QCSE is typically within 20 meV,^18,35–37^ consistent with the shifts observed in Figure 5c. However, spectral diffusion driven by fluctuations in the local electric field can span up to 50 meV, making it difficult to clearly resolve the QCSE-induced shift. This limitation could be addressed by using higher-quality Qdots with reduced spectral diffusion at room temperature or by applying higher electric fields to enhance the Stark shift beyond the diffusion range. The latter approach, however, is technically challenging as the structures become increasingly vulnerable to damage under stronger electric fields. Another possible approach to address this issue is to perform similar measurements under cryogenic conditions, which would substantially suppress phonon-related broadening and environmental charge fluctuations, leading to reduced spectral diffusion and improved visibility of QCSE tuning. However, an important consideration arises under cryogenic operation. While the QD line width is expected to narrow due to reduced phonon interactions and spectral diffusion, the line width of the plasmonic cavity is comparatively insensitive to temperature. This disparity can reduce the spectral overlap between the QD emission and the plasmonic-cavity mode, which may, in turn, decrease the effective light–matter coupling strength. Therefore, cryogenic operation alone does not provide a straightforward solution to this limitation.

In summary, we demonstrate an on-chip platform for achieving strong light–matter interaction between single or a few Qdots and a plasmonic slit cavity. Precise spatial positioning of Qdots at the cavity hot spot is realized using a DEP process integrated with real-time PL feedback. The coupled structures exhibit clear spectral splitting in PL, consistent with predictions of a quantum model assuming coupling of a two-level system with a single mode. By precharacterizing the plasmonic-cavity properties, we reliably extract coupling strengths for a multitude of fabricated devices. Our analysis reveals variations in coupling strength across different hybrid structures, which are likely determined by the number of emitters coupled to the cavity mode. Furthermore, we explore electrical control over the Qdot resonance via QCSE enabled by the integrated electrodes of the lateral device architecture. While applied electric fields modulate the PL intensity, spectral diffusion at room temperature obscures clear observation of Stark shifts. These results highlight the potential of deterministic, ultracompact, and electrically tunable quantum plasmonic systems for applications in quantum information processing, tunable photonic devices, and an integrable on-chip quantum light source.

## Methods

### Sample Preparation

Monocrystalline gold flakes are synthesized in solution following established protocols.^38–40^ Flakes with a suitable thicknesses are selected based on transmission measurements and transferred onto a substrate prepatterned with gold electrodes using a standard optical lift-off process (Figure S1a). A gallium-focused ion beam (Ga-FIB) is then used to isolate individual electrodes and define a large optical window along with coarse structural features (Figure S1b). High-precision slit cavities and tip gaps are subsequently fabricated using helium ion milling (Orion nanoFab, Zeiss).

Commercial colloidal semiconductor quantum dots (CdSeTe/ZnS Qdot 705 ITK Carboxyl, Q21361MP, Thermo Fisher Scientific) are used as quantum emitters in our experiments. The Qdot solution is diluted in Milli-Q water to achieve appropriate concentrations for different measurements. The aqueous solution was carefully prepared to ensure a uniform distribution of quantum dots across the glass coverslip, avoiding any interaction between different quantum dots. For basic characterization, a 1:6000 dilution is spin-coated onto cleaned microscope coverslips (Gerhard Menzel GmbH). For DEP positioning, a more dilute solution (1:10000) is used.

### Optical Setup

The PL measurement setup is sketched in Figure S2. A 532 nm continuous wave laser (AIST-NT ROU006) serves as the excitation source and is focused onto the sample by using a high-numerical aperture objective (Nikon CFI P-Apo 100×, NA 1.45). For characterizing single-Qdot emission and conducting coupling experiments, an excitation power of 1 μW is typically used, while a higher power of 500 μW is applied to probe gold photoluminescence. The emitted PL signal passes through a dichroic mirror and is routed via a flip mirror either into a spectrometer (HORIBA iHR320) equipped with an EMCCD detector (Andor Newton 970p) or toward a time-resolved photon statistics setup. Independent piezoelectric stages control the sample mount and objective, enabling precise alignment of the laser focus with the sample. Time-correlated single-photon counting (TCSPC) measurements are performed by splitting the PL signal with a 50:50 beam splitter and detecting photons using two avalanche photodiodes (APDs, SPCM-AQR). Photon arrival times are recorded using a field-programmable gate array (FPGA, qutools quTAU H+).

## Supplementary Material



## Data Availability

The data generated and analyzed in this study are available from the corresponding author upon reasonable request.

## Notes

FDTD simulations of plasmonic slit cavities, gold photoluminescence fitting procedures, characterization of quantum dots, detailed steps of the DEP process, classical evaluation of coupling strength, quantum modeling and spectral fitting procedures, and additional experiments on electrically connected strongly coupled devices (PDF)
