# Supplementary material for: On-Chip Plasmonic Slit-Cavity Platform for Room-Temperature Strong Coupling with Deterministically Positioned Colloidal Quantum Dots
Source: Nano Lett. 2026 Feb 27;26(11):3710–9. doi: 10.1021/acs.nanolett.5c05910 (PMC13022876; doi:10.1021/acs.nanolett.5c05910)
Supplement: Supplementary file 1 [file nl5c05910_si_001.pdf]

# **Supplementary Information for** On-chip plasmonic slit-cavity platform for room-temperature strong coupling with deterministically positioned colloidal quantum dots

Jin Qin,*†‡ Benedikt Schurr,†‡ Patrick Pertsch,† Daniel Friedrich,† Max Knopf,† Saeid Asgarnezhad-Zorgabad,¶ Lars Meschede, ¶ Daniel D.A. Clarke,¶ Monika Emmerling,† Artur Podhorodecki,§ Ortwin Hess, *¶ and Bert Hecht*†

†Nano-Optics and Biophotonics Group, Experimentelle Physik 5, Physikalisches Institut, Universität Würzburg & and Röntgen Research Center for Complex Material Research, Physics Institute, Am Hubland, Würzburg, D-97074, Germany.

¶School of Physics and CRANN Institute, Trinity College Dublin, Dublin, Ireland.

§Department of Experimental Physics, Wroclaw University of Science and Technology, Wybrzeze Wyspianskiego, Wroclaw, 50-370, Poland.

‡ These two authors contribute equally.

* jin.qin@uni-wuerzburg.de; Ortwin.Hess@tcd.ie; bert.hecht@uni-wuerzburg.de

## S1 Simulations

The quasinormal mode (QNM) analysis of the plasmonic slit cavity was performed using the COMSOL mode solver. The dielectric function of monocrystalline gold was taken from Olmon et al.[1], and a refractive index of 1.52 was used for the glass substrate. Perfectly matched layers were applied to the simulation boundaries to absorb outgoing electromagnetic waves and eliminate artificial reflections. Numerical artifacts were removed to ensure accurate results. Figure 1c was generated by summing the contributions of the second- and third-order QNMs, modeled using Lorentz oscillators.

## S2 Gold PL fitting model

The exponential background in gold PL is consistent with the intrinsic photoluminescence of plasmonic gold nanostructures, which originates from inelastic electronic transitions resulting in photon emission with an extremely low quantum yield (on the order of $10^{-10}$). As a result, resolving this background signal typically requires relatively high excitation powers (500 $\mu$W). Recent theoretical work by Loirette-Pelous and Greffet provides a general framework for metallic photoluminescence in plasmonic systems, demonstrating that such broadband emission can be quantitatively described even under continuous-wave excitation[2].

The observed peak centered near 730 nm is due to the spectral response of the plasmonic slit cavity, which enhances the local density of optical states (LDOS) at this wavelength. Owing to

local field enhancement and the increased local density of optical states associated with the plasmonic mode, the gold PL background develops a distinct peak, which becomes clearly visible at sufficiently high excitation power. In our experiments, this gold PL emission therefore serves as a convenient probe of the cavity resonance in the absence of coupled emitters.

The spectrum of the plasmonic slit cavity is characterized by analyzing the intrinsic linear photoluminescence (PL) of gold, excited using a 532 nm continuous-wave laser diode at an intensity of $4.4 \times 10^{9} \mathrm{W/m^{2}}$[3]. The resonance energy and quality factor Q of the slit cavity resonance are extracted from the gold PL spectrum using a composite fitting function. This model includes an exponential decay to account for the broad unstructured background and a Lorentzian to describe the cavity mode:

$$f(\omega) = \frac{A_a e^{-b\omega}}{\gamma_a[1+4(\omega-\omega_a)^2/\gamma_a^2]} + A_a e^{-b\omega} + c \quad \text{(S1)}$$

Here, $A_a$, $\gamma_a$, and $\omega_a$ represent the amplitude, full width at half maximum (FWHM), and resonance frequency of the slit cavity, respectively. The extracted quality factor is 15.8, which closely matches the value obtained from simulations 20.

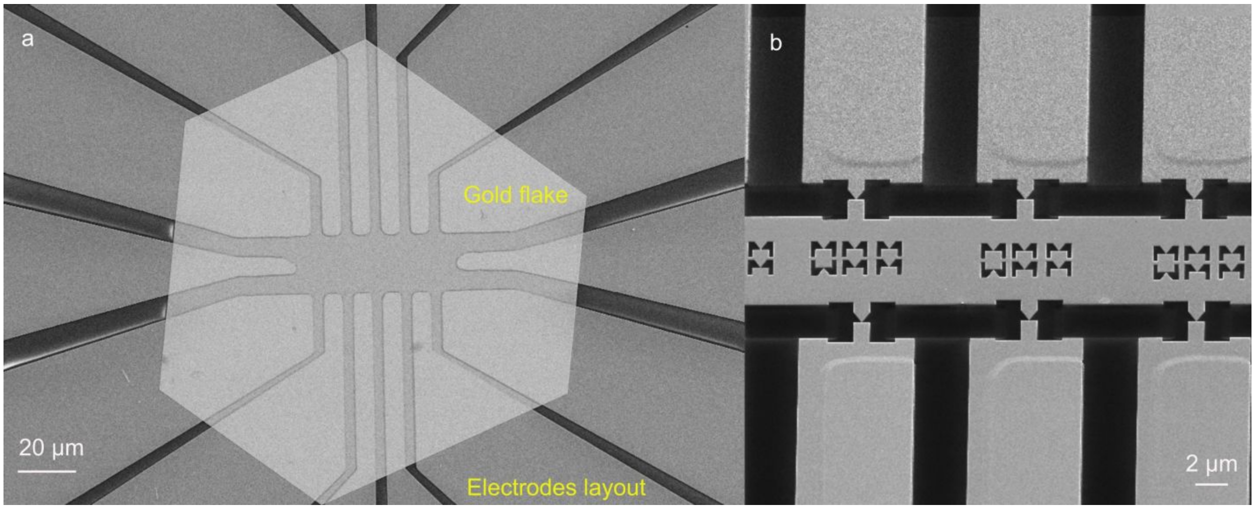


**Figure S1 SEM images of the fabrication process for the on-chip plasmonic device.** **a**, A monocrystalline gold flake is transferred onto the pre-patterned electrode layout. **b**, Gallium FIB processing is used to electrically isolate the electrode fingers and define the large optical window.

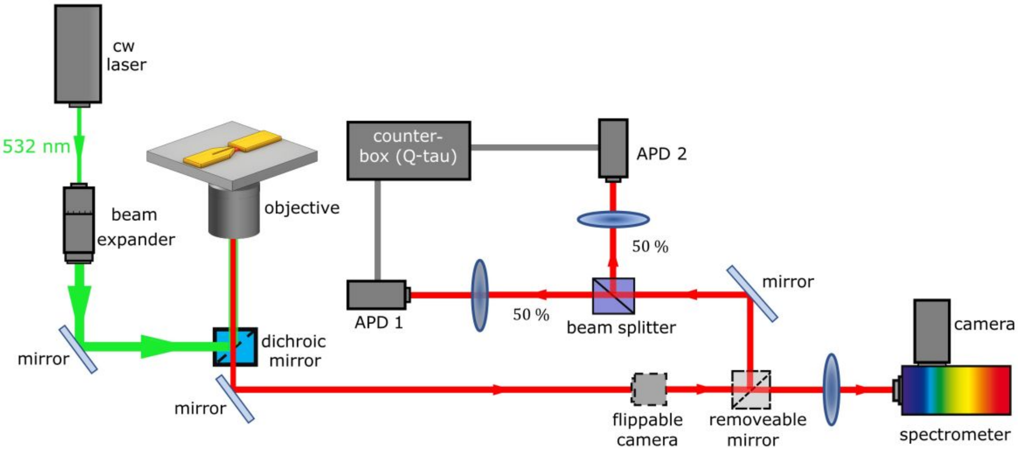


**Figure S2 Schematic of PL measurement setup.**

## S3 Quantum Dot

The unnormalized photon correlation figure and the corresponding uncertainty calculation are shown in Figure S4a-b. The uncertainty in the correlation function is calculated using the same approach described in this literature[4]. Specifically, the uncertainty is given by:

$$\Delta g^{(2)}(\tau) = \frac{\sqrt{M}}{I_A I_B w T}$$

where $M$ is the number of coincidence counts recorded per time bin, $I_A$ and $I_B$ are the time-averaged count rates of the two detection channels, $w$ is the time-bin width, and $T$ is the total acquisition time. In our measurements, $I_A \approx I_B \approx 35$kcounts/s, the time-bin width is $w = 0.81$ns, and the total acquisition time is $T = 240$s. Using these parameters, we calculate the uncertainty of $g^{(2)}(\tau)$ for each delay bin based on the measured coincidence counts.

To verify the presence of clusters in the Qdot aqueous solution, we spin-coated the solution onto a coverslip and performed AFM scans, as shown in Figure S4. Only a small fraction of the features corresponds to individual Qdots, which exhibit small dimensions comparable to the one in Figure 2a. In contrast, some features display larger dimensions, indicating that they are likely composed of multiple Qdots. The exact number of QDots within a cluster can be further characterized using photon statistics measurements. As presented in Figure S5c–e, we show three representative $g^{(2)}(\tau)$ measurements corresponding to effective emitter numbers ranging from one to three. From the $g^{(2)}(\tau)$ measurement, the lifetime of Qdot $\tau_{qd}$ can also be extracted by fitting the correlation curve by using $g^{(2)}(\tau) = \frac{N-1}{N} + \frac{1-e^{-|\tau|/\tau_{qd}}}{N}$, where $N$ is number of photon emitter.

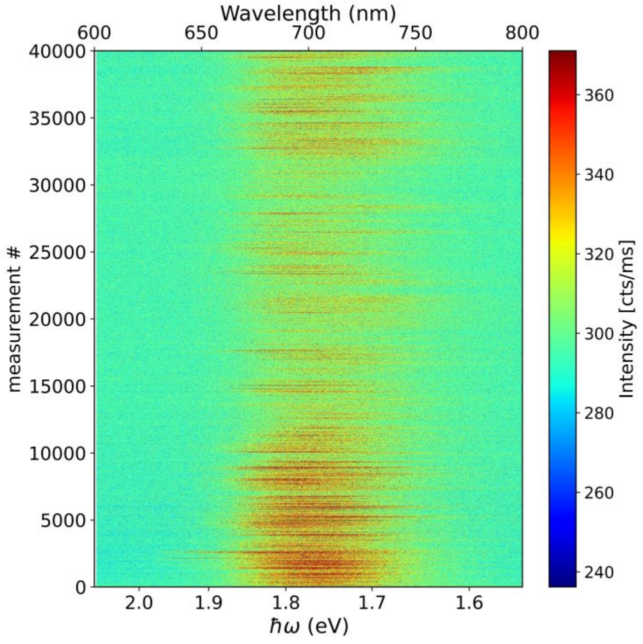


**Figure S3 Time traced PL spectra from a single Qdot.** Each spectrum is taken within an integration time of 33 ms.

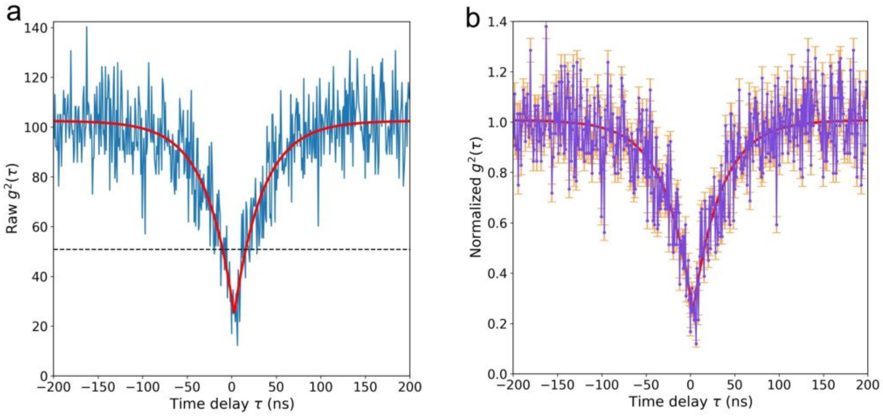


**Figure S4. a,** The unnormalized $g^{(2)}(\tau)$ data and **b**, the corresponding uncertainty evaluation.

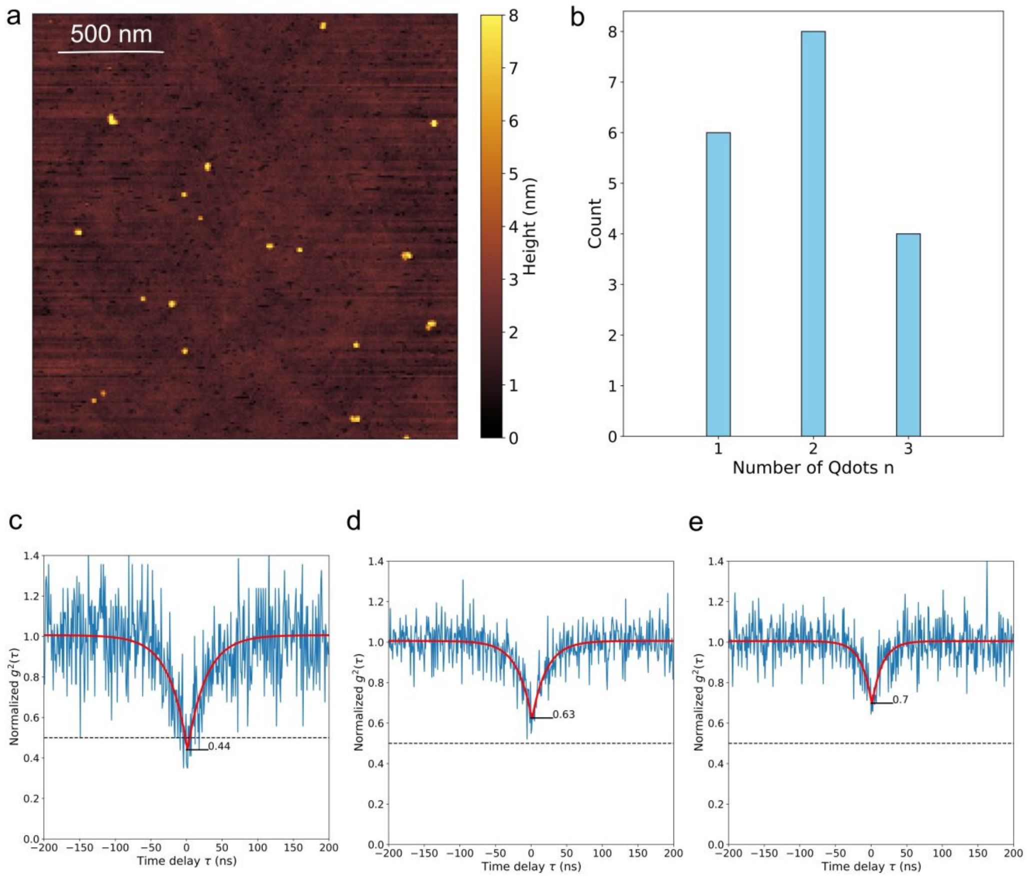


**Figure S5 AFM scans of Qdots spin-coated on a coverslip. a**, AFM image over an area of 2 μm by 2 μm. **b**, Statistics of the number of Qdots per cluster. **c-e**, Three representative $g^{(2)}(\tau)$ measurements corresponding to effective emitter numbers ranging from one to three

## S4 Dielectrophoresis process

Dielectrophoresis (DEP) occurs when a polarizable particle is placed in a spatially non-uniform electric field, resulting in a net force due to the field gradient. The time-averaged DEP force acting on a spherical particle can be expressed as[5]:

$$\langle F_{DEP} \rangle = 2\pi\epsilon_1 R^3 [\frac{\epsilon_2-\epsilon_1}{\epsilon_2+2\epsilon_1} + \frac{3(\epsilon_1\sigma_2-\epsilon_2\sigma_1)}{\tau_m(\sigma_2+2\sigma_1)^2(1+\omega^2\tau_m^2)^2}] \nabla E_{rms}^2 \quad \text{(S2)}$$

where $\epsilon_1$ ($\epsilon_2$) and $\sigma_1$ ($\sigma_2$) denote the permittivity and conductivity of the surrounding medium (particle), respectively. R is the particle radius, $\omega$ is the frequency of the applied field, and $E_{rms}$ is the root-mean-square magnitude of the electric field. The term $\tau_m = \frac{\epsilon_2+\epsilon_1}{\sigma_2+2\sigma_1}$ is the Maxwell-Wagner relaxation time, characterizing the decay of the dipolar charge distribution at the particle interface.

Equation S2 indicates that the magnitude of the DEP force can be tuned by adjusting the frequency $\omega$, providing a route to optimize the trapping process. If $\langle F_{DEP} \rangle$ is too large, many particles are pulled toward the target, promoting clustering. Increasing $\omega$ reduces the force, allowing more controlled placement of particles—especially when combined with real-time feedback.

The detailed DEP procedure is illustrated in Figure S6. First, the Qdot solution is ultrasonicated for one minute to disperse aggregates, then diluted in Milli-Q water at a 1:10000 ratio, followed by another one-minute ultrasonication. A 5 μL droplet is deposited on the structured gold flake, fully covering the active area. Two micromanipulators contact the electrodes, which are connected to a function generator (DS345, Stanford Research Systems). A 532 nm green laser is focused onto the slit cavity, and the PL signal is monitored in real-time using a sensitive avalanche photodiode (APD).

Upon applying an AC electric field, Qdots are drawn to the cavity gap. A sudden increase in photon counts indicates successful capture, triggering immediate shutdown of the function generator to prevent additional accumulation. Importantly, when the function generator is turned off, the DEP force immediately vanishes. Due to the overdamped nature of the system, the QDots

do not continue to accelerate; instead, their velocity relaxes essentially instantaneously, and only Brownian diffusion remains unless they have already attached to the surface. Correct positioning is further verified through real-time PL feedback: the applied field is halted once the PL signature indicates that a single Qddot or a small cluster have reached the cavity hotspot. This feedback-assisted control ensures that only a single QDot or a small cluster is positioned, effectively preventing the accumulation of multiple QDots or contaminants, as illustrated in Figure 3f. After positioning, further motion of Qdot is suppressed by strong geometric confinement and surface interactions, including adsorption to the slit sidewalls, which stabilize the Qdot position within the cavity. The laser spot and micromanipulators are then moved to the next cavity, and the process is repeated. To avoid drying-up, water is added from time to time. After the DEP process is complete, the droplet is rinsed off with Milli-Q water followed by ethanol, and the sample is then thoroughly dried.

Owing to the real-time PL feedback employed during the DEP process, the positioning procedure is highly repeatable. Once all DEP parameters are optimized, an AC voltage is applied with real-time PL feedback. In combination with these optimized DEP conditions, the QD solution is adjusted to a relatively low particle density of QDs and clusters. Together, these measures yield a success rate of approximately 50% for attracting single QDs or small clusters, as evidenced by the presence of small objects attached to the on-chip nanoslit tip in roughly half of the AFM scans. In the remaining 50% of all cases, either no material is attracted—indicated by the absence of both PL signals and AFM-detectable features—or excessive material is collected, resulting in larger aggregates. These aggregates consist of CdSeTe/ZnS clusters, reflecting the inherently statistical

nature of the DEP process. While this yield of about 50% is sufficient for studies involving individual junctions, it remains insufficient for future studies which may require several junctions to be filled. Nevertheless, the success rate can be further improved through further optimization of the DEP parameters and Qdot solution density, as well as by reducing contaminants in the Qdot solution and enhancing the reliability of the PL feedback mechanism.

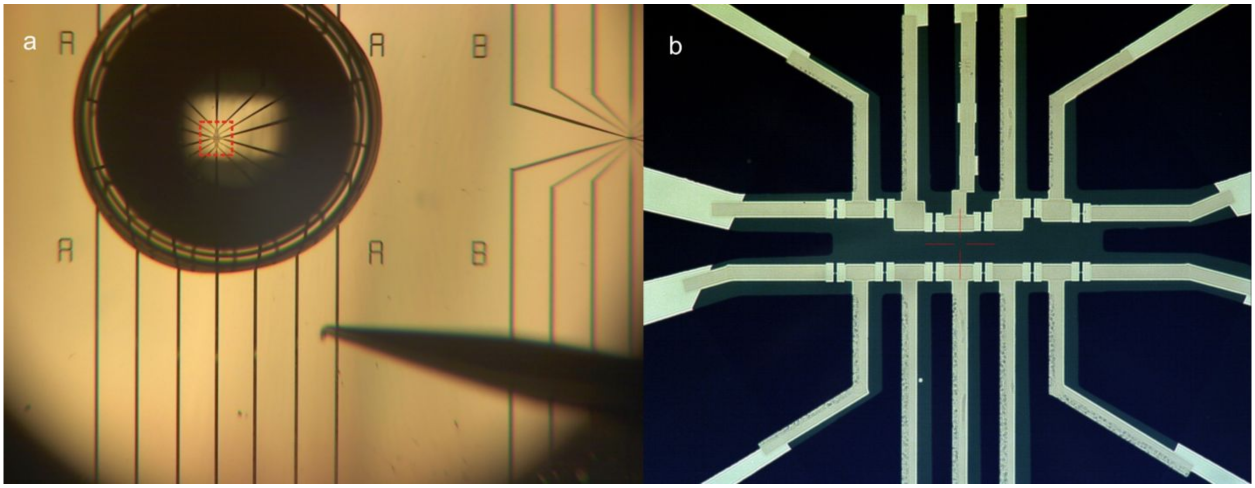


**Figure S6 Microscopic images of the DEP process. a**, A 5 μL aqueous droplet containing Qdots is placed on top of the structures. Micromanipulators are used to contact the two electrodes from above, while PL spectra are monitored from below using a high-NA objective. **b**, A zoomed-in view showing the transmission image of the structured gold flake under the droplet (corresponding to the red dashed box in **a**).

**S5 Classical evaluation**

FDTD simulations were performed to investigate the coupled states between a plasmonic slit cavity and a single quantum emitter. The dielectric function of single-crystalline gold was taken

from Olmon et al.[1]. To model the quantum dot exciton state, its permittivity was described by a Lorentzian function,

$$\varepsilon_{Qdot}(\omega) = \varepsilon_\infty + f\omega_0^2/(\omega_0^2 - \omega^2 - i\gamma_0\omega) \tag{S3}$$

following the Refence[6]. Here, $\varepsilon_\infty$ is the high-frequency contribution of the CdSe/ZnS quantum dot matrix dielectric function, with a value of 5. The oscillator strength was set to 0.3, the lowest excitonic transition was placed at 1.76 eV, and the exciton linewidth was 140 meV.

The coupling energy g between the plasmonic slit cavity and the quantum dot is given by the scalar product of the quantum dot dipole moment $\mu$ and the vacuum field amplitude $E_0$ at position $\boldsymbol{r}$,

$$E_0(\boldsymbol{r}) = \sqrt{\frac{\hbar\omega}{2\varepsilon_0 V_{eff}(\boldsymbol{r})}} \tag{S4}$$

where $\mu$ is the dipole moment of the quantum dot, $\hbar\omega$ is the photon energy, $\varepsilon_0$ is the permittivity of free space, and $V_{eff}(\boldsymbol{r})$ is the effective mode volume of the slit cavity. The effective mode volume was calculated using quasi-normal modes (QNMs) with complex frequencies following Sauvan et al.[3,7]. Based on these simulations, the single-emitter coupling strength is estimated to be 72.8 meV.

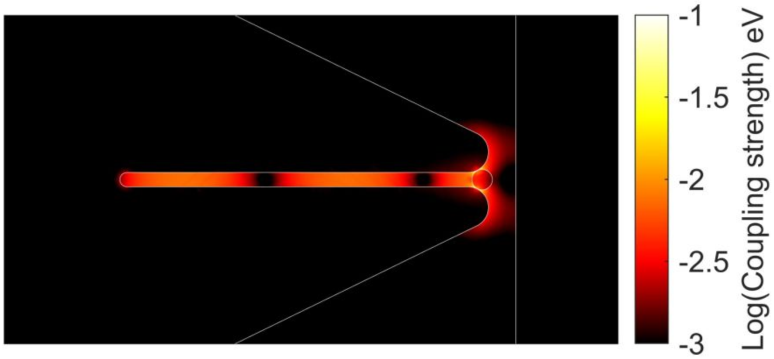

**Figure S7 Map of the coupling strength g in eV (log scale) obtained from FDTD simulations.** The quantum dot is assigned with a dipole moment of 9 Debye. To obtain the spatially dependent coupling strength, the effective mode volume was multiplied by a dimensionless overlap factor $\eta = \max\left(\left|\vec{E}\right|^2\right)/\left|\vec{E}\right|^2$. Scale bar: 50 nm.

**S6 Quantum model**

Light–matter strong coupling can be described by the Jaynes–Cummings Hamiltonian (with $\hbar = 1$)[8]:

$$H = \omega_{cav}a^{\dagger}a + \omega_{qd}\sigma^{\dagger}\sigma + g(a^{\dagger}\sigma + \sigma^{\dagger}a) \qquad \text{S5}$$

where $a$ ($a^{\dagger}$) are the annihilation (creation) operators of a single bosonic cavity mode with energy $\omega_{cav}$, and $\sigma^{\dagger}(\sigma)$ are the raising (lowering) operators of a two-level system with transition energy $\omega_{qd}$. The two systems—the optical cavity and Qdot—are coupled with strength g.

To analyze the dynamics of this hybrid system, we solve the master equation for the density matrix $\rho$:

$$\frac{d\rho}{dt} = -i[H,\rho] + \sum_i \mathcal{L}_i(\rho) \qquad \text{S6}$$

where $\mathcal{L}_i$ are Lindblad superoperators representing various dissipative processes, including radiative decay ($\gamma_{cav}$, $\gamma_{qd}$), incoherent pumping ($P_{cav}$, $P_{qd}$), and pure dephasing ($\gamma_{\phi}$). These are expressed as:

$$\sum_i \mathcal{L}_i(\rho) = \frac{\gamma_{cav}}{2}\left(2a\rho a^{\dagger} - \{a^{\dagger}a,\rho\}\right) + \frac{\gamma_{qd}}{2}\left(2\sigma\rho\sigma^{\dagger} - \{\sigma^{\dagger}\sigma,\rho\}\right) +$$

$$\frac{P_{cav}}{2}\left(2a^{\dagger}\rho a-\{aa^{\dagger},\rho\}\right)+\frac{P_{qd}}{2}\left(2\sigma^{\dagger}\rho\sigma-\{\sigma\sigma^{\dagger},\rho\}\right)+$$

$$\frac{\gamma_{\phi}}{2}(\sigma_z\rho\sigma_z-\rho). \qquad \text{S7}$$

We assume that cavity emission dominates in the hybrid system, allowing us to neglect direct emission from the quantum dot[9]. Under this assumption, the emission spectrum $S(\omega)$ is calculated as:

$$S(\omega)\propto\Re\left(\int_0^{\infty}\langle a^{\dagger}(\tau)a(0)\rangle e^{-i\omega\tau}d\tau\right) \qquad \text{S8}$$

Numerical simulations are performed using the QuTiP (Quantum Toolbox in Python) package[10].

**S7 Spectral fitting procedure**

Since the bare slit cavity is characterized prior to the DEP process, the cavity resonance frequency $\omega_{cav}$ and cavity loss rate $\gamma_{cav}$ can be extracted by fitting the intrinsic gold PL spectrum, as shown in Figure 1c. These parameters are kept fixed during the fitting of the coupled spectra. The Qdot resonance frequency $\omega_{qd}$, however, varies due to spectral diffusion at room temperature and is adjusted accordingly during the fitting procedure.

The total linewidth of the Qdot emission consists of two contributions: the radiative decay rate $\gamma_{qd}$ and the pure dephasing rate $\gamma_{\phi}$. From time-resolved photon statistics measurements, the Qdot lifetime $\tau$ is determined to be 33 ns, which corresponds to a radiative decay rate of $\gamma_{qd}$ $=20neV$, based on the relation $\gamma_{\sigma}\tau=\hbar$. The dominant contribution to the broad linewidth of the Qdot arises from pure dephasing ($\gamma_{\phi}$), which fluctuates over time as shown in Figure 2d.

The detailed fitting parameters used in the simulations and spectral fitting are summarized in Tables S1, S2, and S3.

**Table S1 Fitting parameters for Figure 4a with quantum model.**

| $\boldsymbol{\omega_{cav}}$ (eV) | g(meV) | $\boldsymbol{\omega_{qd}}$(eV) | $\boldsymbol{\gamma_{cav}}$ (meV) | $\boldsymbol{P_{cav}}$(meV) | $\boldsymbol{\gamma_{qd}}$(neV) | $\boldsymbol{P_{qd}}$ (meV) | $\boldsymbol{\gamma_{\phi}}$ (meV) |
|---|---|---|---|---|---|---|---|
| 1.69 | 71.0 | 1.73 | 128.9 | 17.8 | 20.0 | 0.16 | 55.7 |
| 1.69 | 73.0 | 1.745 | 128.9 | 10.1 | 20.0 | 5.0 | 55.7 |
| 1.69 | 73.0 | 1.73 | 128.9 | 20.7 | 20.0 | 4.4 | 55.7 |
| 1.69 | 76.0 | 1.745 | 128.9 | 5.1 | 20.0 | 8.5 | 64.0 |
| 1.69 | 69.0 | 1.735 | 128.9 | 12.8 | 20.0 | 3.9 | 50.9 |

**Table S2 Fitting parameters for Figure 4b with quantum model.**

| $\boldsymbol{\omega_{cav}}$ (eV) | g(meV) | $\boldsymbol{\omega_{qd}}$(eV) | $\boldsymbol{\gamma_{cav}}$ (meV) | $\boldsymbol{P_{cav}}$(meV) | $\boldsymbol{\gamma_{qd}}$(neV) | $\boldsymbol{P_{qd}}$ (meV) | $\boldsymbol{\gamma_{\phi}}$ (meV) |
|---|---|---|---|---|---|---|---|
| 1.75 | 98.0 | 1.78 | 206.9 | 38.8 | 20.0 | 0 | 71.0 |
| 1.75 | 100.0 | 1.785 | 206.9 | 23.9 | 20.0 | 4.2 | 77.0 |
| 1.75 | 96.0 | 1.79 | 206.9 | 37.2 | 20.0 | 0 | 55.7 |
| 1.75 | 104.0 | 1.8 | 206.9 | 27.7 | 20.0 | 9.3 | 55.7 |
| 1.75 | 105.0 | 1.795 | 206.9 | 38.7 | 20.0 | 0 | 66.5 |

**Table S3 Fitting parameters for Figure 4c with quantum model.**

| $\omega_{cav}$ (eV) | g(meV) | $\omega_{qd}$(eV) | $\gamma_{cav}$ (meV) | $P_{cav}$(meV) | $\gamma_{qd}$(neV) | $P_{qd}$ (meV) | $\gamma_{\phi}$ (meV) |
|---|---|---|---|---|---|---|---|
| 1.71 | 121.0 | 1.76 | 109.8 | 11.1 | 20.0 | 11.2 | 23.9 |
| 1.71 | 123.0 | 1.755 | 109.8 | 3.9 | 20.0 | 18.0 | 49.2 |
| 1.71 | 126.0 | 1.76 | 109.8 | 0.1 | 20.0 | 8.5 | 32.9 |
| 1.71 | 120.0 | 1.755 | 109.8 | 17.0 | 20.0 | 7.4 | 28.2 |
| 1.71 | 125.0 | 1.75 | 109.8 | 4.9 | 20.0 | 10.9 | 55.7 |

In Figure S8a–c, we present additional AFM scans of coupled devices fabricated using the same DEP process with feedback control. Typically, AFM characterization is performed after spectral splitting is observed in the PL measurements, serving as further confirmation of the coupled behavior. Furthermore, Figure S8d shows coupling strengths extracted from PL spectra of ten different coupled structures. While some fluctuations are observed across devices, these can be explained by variations in the dipole orientation of the Qdots and by differences in the number of Qdots coupled to the plasmonic slit cavity mode.

## S8 Polariton dynamics

When a quantum emitter is coupled to an optical cavity, an enhanced local density of optical states can increase the spontaneous emission rate, typically resulting in a reduced emitter lifetime. This regime is commonly referred to as the weak-coupling regime, and such lifetime shortening has been extensively reported experimentally[11,12].

However, when the coupling strength between the quantum emitter and the cavity becomes sufficiently large, the system enters the strong-coupling regime. In this regime, excitonic and photonic states hybridize to form new eigenstates known as polaritons. These hybrid light--matter states split into a lower polariton (LP) and an upper polariton (UP), each being a coherent linear superposition of the exciton and cavity photon modes.

The excitonic and photonic fractions of the polariton states are quantified by the Hopfield coefficients $|X|^2$ and $|C|^2$, respectively[13], which are given by:

$$|X|^2 = \frac{1}{2}\left(1 + \frac{\Delta\omega}{\sqrt{\Delta\omega^2 + 4g^2}}\right)$$

$$|C|^2 = \frac{1}{2}\left(1 - \frac{\Delta\omega}{\sqrt{\Delta\omega^2 + 4g^2}}\right)$$

where $\Delta\omega$ denotes the energy detuning between the cavity resonance and the two-level emitter, and $g$ is the light-matter coupling strength.

The decay rates of the lower and upper polariton states, $\gamma_{LP}$ and $\gamma_{UP}$, are determined by the corresponding excitonic and photonic fractions and can be expressed as:

$$\gamma_{LP} = |X|^2\gamma_{QD} + |C|^2\gamma_{cav}$$

$$\gamma_{UP} = |C|^2\gamma_{QD} + |X|^2\gamma_{cav}$$

where $\gamma_{QD}$ and $\gamma_{cav}$ are the intrinsic decay rates of the quantum dot and the cavity mode, respectively.

When the cavity and emitter are on resonance $\Delta\omega = 0$), both polariton states have identical decay rates that simplify to $(\gamma_{QD} + \gamma_a)/2$. In our experiment, the plasmonic cavity exhibits significantly higher losses compared to the quantum emitter, such that the polariton decay is

dominated by the cavity contribution. Consequently, the polariton lifetimes are on the order of tens of femtoseconds.

Resolving such ultrashort lifetimes is not practical with conventional time-resolved detection techniques based on photodiodes or time-correlated single-photon counting, due to their limited temporal bandwidth. Similarly, performing a $g^{(2)}(\tau)$ measurement on the strongly coupled device is not feasible in this regime, as the available time-bin resolution is much larger than the polariton lifetime and therefore insufficient to resolve antibunching features associated with polaritonic emission.

**S9 Another dataset for an electrically connected strongly coupled device**

Another demonstration that spectral diffusion plays a dominant role in QCSE experiments at room temperature is provided in Figure S9a-b, where we perform a voltage sweep sequence: starting from zero, increasing to a positive field, returning to zero, and then applying a negative field, as illustrated in Figure S9a. The corresponding PL intensities and extracted Qdot resonances are shown in Figure S9b. A significant drop in PL intensity is observed under a positive applied electric field, which is partially recovered upon returning to zero voltage. Conversely, under a negative applied field, the PL intensity remains relatively stable. This asymmetric response can likely be attributed to the presence of a local internal electric field $E_{int}$, as schematically depicted in FigureS9c. When $E_{applied}$ and $E_{int}$ are in the same direction, the total field increases, further reducing electron-hole wavefunction overlaps and decreasing PL intensity. In contrast, when the two fields are oppositely directed, the effective field is reduced or even canceled, potentially

neutralizing the Qdot and enhancing PL emission. Particularly, the PL intensities at zero applied voltage (measurements #0, #2, #4, #6, and #8) exhibit significant fluctuations, indicating that the internal electric field $E_{int}$ varies randomly over time and can be comparable in magnitude to the externally applied field $E_{int}$[13,14]. Additionally, the resonance tunability remains difficult to quantify, as the spectral fluctuations observed at zero applied field are larger than those induced by the applied voltage, further obscuring clear Stark shifts.

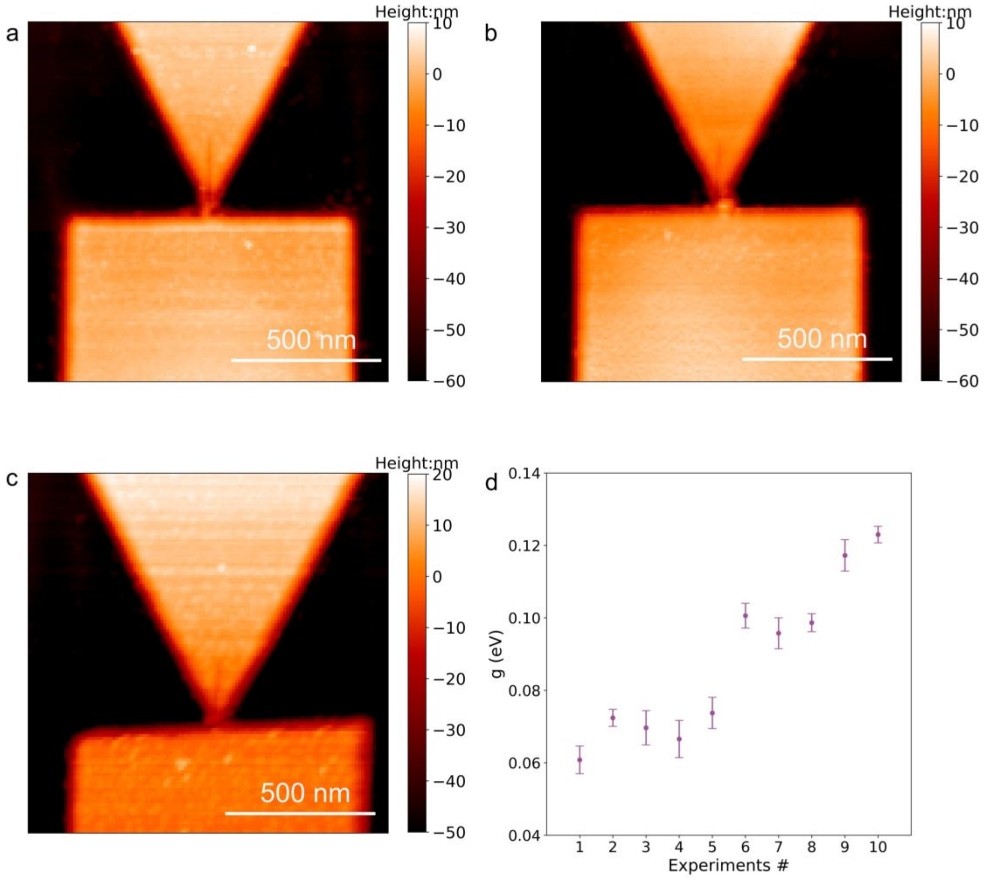


**Figure S8 AFM Characterization and Coupling Strength Statistics of Coupled Devices**. **a–c**, AFM scans of three additional coupled devices. **d**, Coupling strengths extracted from ten other strongly coupled devices.

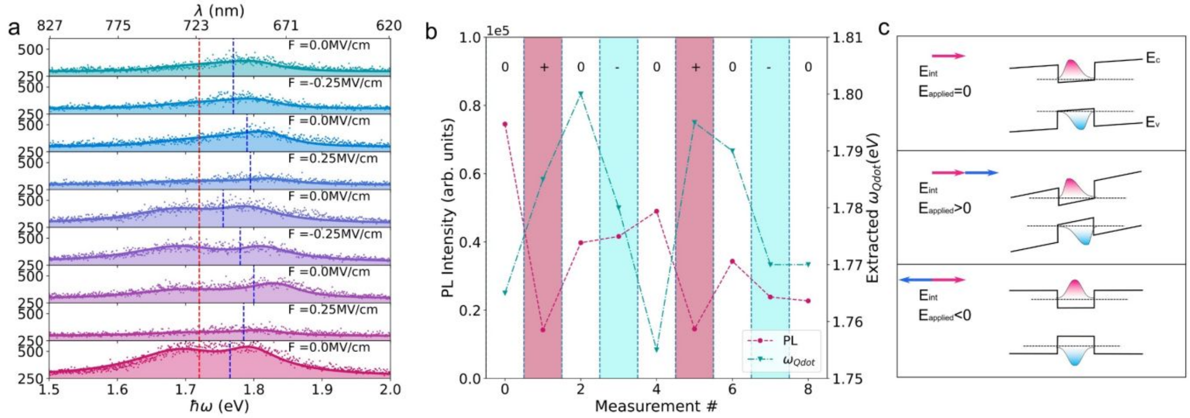


**Figure S9 Additional split PL spectra with different applied voltages. a**, Recorded split PL spectra using a voltage sequence of zero, positive, zero, and negative electric fields. **b**, Corresponding PL intensities and extracted Qdot resonances as a function of the applied electric field. **c**, Band diagrams of the Qdot showing electron and hole wavefunction overlap under the influence of the $E_{applied}$ and $E_{int}$. In the top panel, $E_{applied} = 0$, and the fluctuating $E_{int}$ causes spectral diffusion with varied PL intensities. In the middle and bottom panels, the direction of $E_{applied}$ is reversed, leading to different effective fields and thus varying PL intensities due to changes in wavefunction overlap.

## Reference


(1) Olmon, R. L.; Slovick, B.; Johnson, T. W.; Shelton, D.; Oh, S.-H.; Boreman, G. D.; Raschke, M. B. Optical Dielectric Function of Gold. *Phys. Rev. B* **2012**, *86* (23), 235147. https://doi.org/10.1103/PhysRevB.86.235147.

(2) Loirette-Pelous, A.; Greffet, J.-J. Theory of Photoluminescence by Metallic Structures. *ACS Nano* **2024**, *18* (46), 31823–31833. https://doi.org/10.1021/acsnano.4c07637.

(3) Friedrich, D.; Qin, J.; Schurr, B.; Tufarelli, T.; Groß, H.; Hecht, B. Anticrossing of A Plasmonic Nanoresonator Mode and A Single Quantum Dot at Room Temperature. *Advanced Science* **2025**, *12* (36), e06676. https://doi.org/10.1002/advs.202506676.

(4) Fishman, R. E. K.; Patel, R. N.; Hopper, D. A.; Huang, T.-Y.; Bassett, L. C. Photon-Emission-Correlation Spectroscopy as an Analytical Tool for Solid-State Quantum Defects. *PRX Quantum* **2023**, *4* (1), 010202. https://doi.org/10.1103/PRXQuantum.4.010202.

(5) Jones, T. B. *Electromechanics of Particles*; Cambridge University Press: Cambridge, 1995. https://doi.org/10.1017/CBO9780511574498.

(6) Schlather, A. E.; Large, N.; Urban, A. S.; Nordlander, P.; Halas, N. J. Near-Field Mediated Plexcitonic Coupling and Giant Rabi Splitting in Individual Metallic Dimers. *Nano Lett.* **2013**, *13* (7), 3281–3286. https://doi.org/10.1021/nl4014887.

(7) Sauvan, C.; Hugonin, J. P.; Maksymov, I. S.; Lalanne, P. Theory of the Spontaneous Optical Emission of Nanosize Photonic and Plasmon Resonators. *Phys. Rev. Lett.* **2013**, *110* (23), 237401. https://doi.org/10.1103/PhysRevLett.110.237401.

(8) Shore, B. W.; Knight, P. L. The Jaynes-Cummings Model. *Journal of Modern Optics* **1993**, *40* (7), 1195–1238. https://doi.org/10.1080/09500349314551321.

(9) Valle Reboul, E. del. Quantum Electrodynamics with Quantum Dots in Microcavities. PhD Thesis, Universidad Autónoma de Madrid, 2009.

(10) Johansson, J. R.; Nation, P. D.; Nori, F. QuTiP: An Open-Source Python Framework for the Dynamics of Open Quantum Systems. *Computer physics communications* **2012**, *183* (8), 1760–1772.

(11) Hoang, T. B.; Akselrod, G. M.; Mikkelsen, M. H. Ultrafast Room-Temperature Single Photon Emission from Quantum Dots Coupled to Plasmonic Nanocavities. *Nano letters* **2016**, *16* (1), 270–275.

(12) Tran, T. T.; Wang, D.; Xu, Z.-Q.; Yang, A.; Toth, M.; Odom, T. W.; Aharonovich, I. Deterministic Coupling of Quantum Emitters in 2D Materials to Plasmonic Nanocavity Arrays. *Nano Lett.* **2017**, *17* (4), 2634–2639. https://doi.org/10.1021/acs.nanolett.7b00444.

(13) Hopfield, J. J. Theory of the Contribution of Excitons to the Complex Dielectric Constant of Crystals. *Physical Review* **1958**, *112* (5), 1555–1567. https://doi.org/10.1103/PhysRev.112.1555.

(14) Empedocles, S. A.; Norris, D. J.; Bawendi, M. G. Photoluminescence Spectroscopy of Single CdSe Nanocrystallite Quantum Dots. *Phys. Rev. Lett.* **1996**, *77* (18), 3873–3876. https://doi.org/10.1103/PhysRevLett.77.3873.

(15) Conradt, F.; Bezold, V.; Wiechert, V.; Huber, S.; Mecking, S.; Leitenstorfer, A.; Tenne, R. Electric-Field Fluctuations as the Cause of Spectral Instabilities in Colloidal Quantum Dots. *Nano Lett.* **2023**, *23* (21), 9753–9759. https://doi.org/10.1021/acs.nanolett.3c02318.
